# 5-Chloro­acetyl-4-methyl-2,3,4,5-tetra­hydro-1*H*-1,5-benzodiazepin-2-one

**DOI:** 10.1107/S1600536809034813

**Published:** 2009-09-05

**Authors:** K. Ravichandran, P. Sakthivel, S. Ponnuswamy, P. Ramesh, M. N. Ponnuswamy

**Affiliations:** a Centre of Advanced Study in Crystallography and Biophysics, University of Madras, Guindy Campus, Chennai 600 025, India; b Department of Chemistry, Government Arts College (Autonomous), Coimbatore 641 018, India

## Abstract

In the title compound, C_12_H_13_ClN_2_O_2_, the benzodiazepine ring adopts a distorted boat conformation. The carbonyl O atom and the Cl atom of the chloro­acetyl group are in a *cis* conformation. The crystal packing is controlled by inter­molecular C—H⋯O and N—H⋯O inter­actions.

## Related literature

For hydrogen-bond motifs, see: Bernstein *et al.* (1995 ▶). For puckering and asymmetry parameters, see: Cremer & Pople (1975 ▶); Nardelli (1983 ▶). For the use of benzodiazepines in the treatment of gastrointestinal and central nervous system disorders, see: Rahbaek *et al.* (1999 ▶).
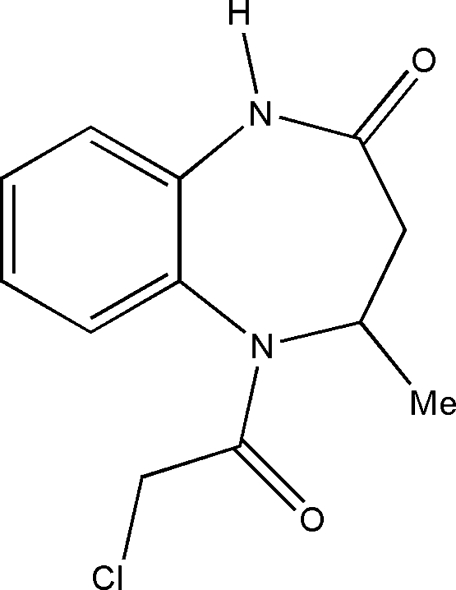


## Experimental

### 

#### Crystal data

C_12_H_13_ClN_2_O_2_
*M* *_r_* = 252.69
Monoclinic, 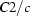

*a* = 16.7656 (4) Å
*b* = 8.8171 (2) Å
*c* = 17.0125 (4) Å
β = 105.803 (1)°
*V* = 2419.80 (10) Å^3^
*Z* = 8
Mo *K*α radiation
μ = 0.31 mm^−1^
*T* = 293 K
0.30 × 0.25 × 0.20 mm

#### Data collection

Bruker Kappa APEXII area-detector diffractometer
Absorption correction: multi-scan (*SADABS*; Sheldrick, 2001 ▶) *T*_min_ = 0.912, *T*_max_ = 0.940
17051 measured reflections
4087 independent reflections
2835 reflections with *I* > 2σ(*I*)
*R*_int_ = 0.026

#### Refinement

*R*[*F* ^2^ > 2σ(*F* ^2^)] = 0.043
*wR*(*F* ^2^) = 0.128
*S* = 1.02
4087 reflections
159 parameters
H atoms treated by a mixture of independent and constrained refinement
Δρ_max_ = 0.30 e Å^−3^
Δρ_min_ = −0.33 e Å^−3^

### 

Data collection: *APEX2* (Bruker, 2004 ▶); cell refinement: *SAINT* (Bruker, 2004 ▶); data reduction: *SAINT*; program(s) used to solve structure: *SHELXS97* (Sheldrick, 2008 ▶); program(s) used to refine structure: *SHELXL97* (Sheldrick, 2008 ▶); molecular graphics: *ORTEP-3* (Farrugia, 1997 ▶); software used to prepare material for publication: *SHELXL97* and *PLATON* (Spek, 2009 ▶).

## Supplementary Material

Crystal structure: contains datablocks global, I. DOI: 10.1107/S1600536809034813/bt5035sup1.cif

Structure factors: contains datablocks I. DOI: 10.1107/S1600536809034813/bt5035Isup2.hkl

Additional supplementary materials:  crystallographic information; 3D view; checkCIF report

## Figures and Tables

**Table 1:** Hydrogen-bond geometry (Å, °)

*D*—H⋯*A*	*D*—H	H⋯*A*	*D*⋯*A*	*D*—H⋯*A*
C4—H4⋯O2	0.98	2.32	2.6952 (17)	102
N1—H1⋯O1^i^	0.881 (18)	1.958 (18)	2.8375 (16)	176.4 (16)
C7—H7⋯O2^ii^	0.93	2.43	3.2818 (17)	153
C14—H14*A*⋯O1^iii^	0.97	2.52	3.2411 (18)	131

Symmetry codes: (i) 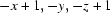; (ii) 
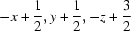
; (iii) 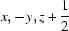.

**Fig. 1.**
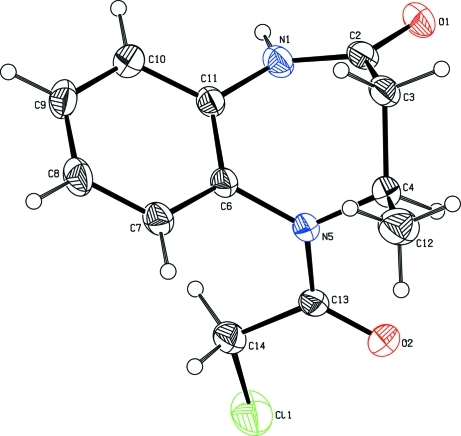
Perspective view of the molecule showing the thermal ellipsoids are drawn at 50% probability level.

**Fig. 2.**
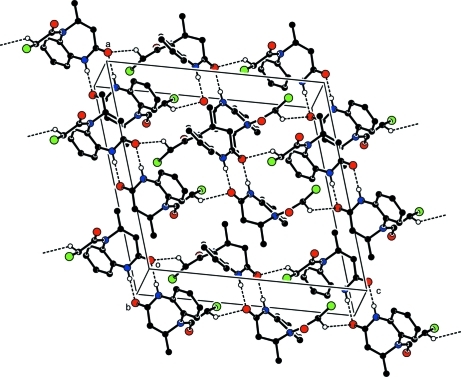
The crystal packing of the molecules viewed down b–axis. H atoms not involved in hydrogen bonding have been omitted for clarity.

## supplementary crystallographic information

### Comment

Benzodiazepines are known for their natural occurrence in filamentous fungi and actinomycetes of the *genera pencillium, aspergillus* and *streptomyces*. Benzodiazepines from *aspergillus* include *asperlicin*, which is used for treatment of gastrointestinal and central nervous system (*CNS*) disorders (Rahbaek *et al.*,1999). In view of these importance and to ascertain the molecular conformation, crystallographic study of the title compound has been carried out.

The *ORTEP* diagram of the title compound is shown in Fig.1. The benzodiazepine ring adopts a distorted boat conformation. The puckering parameters (Cremer & Pople, 1975) and the asymmetry parameters (Nardelli, 1983) for this ring are q_2_ = 0.965 (1) Å, q_3_ = 0.155 (1) Å, φ_2_ = 144.0 (1)°, φ_3_ = 11.4 (5)° and Δ2(C4)=7.8 (1)°. The sum of the bond angles at N1(359.4°) and N5(359.99°) of the benzodiazepine ring is in accrdance with *sp*^2^ hybridization. The choloroacetyl group adopts an extended conformation, which is evidenced from the torsion angle N5—C13—C14—Cl1[-161.9 (1)°].

The crystal packing is controlled by C—H···O and N—H···O types of intra and intermolecular interactions in addition to van der Waals forces. Atom N1 at (*x*, *y*, *z*) donates a proton to O1 (-*x* + 1, -*y*, -*z* + 1), which forms a graph set motif of *R*^2^_2_(8) dimer (Bernstein *et al.*, 1995). The intermolecular hydrogen bond C14—H14A···O1 connect the dimers into a C9 one dimensional chain running along c–axis as shown in Fig 2. Thus the two dimensional network is connected by an intermolecular hydrogen bond C7—H7···O2 which leads to a C6 zig–zag chain running along b–axis.

### Experimental

To a solution of tetrahydro-4-methyl-1,5-benzodiazepin-2-one (0.88 g, 5 mmol) in anhydrous benzene (50 ml) was added triethylamine (2.8 ml, 20 mmol) and chloroacetyl chloride (1.59 ml, 20 mmol). The contents were allowed to reflux on a water bath for 6hrs. The reaction mixture was washed with sodium bicarbonate solution (10%), water and dried. The crude mass was crystallized from ethanol.

### Refinement

The H atom bonded to N was freely refined and the other H atoms were positioned geometrically (C—H=0.93–0.98 Å) and allowed to ride on their parent atoms, with 1.5*U*_eq_(C) for methyl H and 1.2 *U*_eq_(C) for other H atoms.

### Crystal data

**Table:**

C_12_H_13_ClN_2_O_2_	*F*(000) = 1056
*M**_r_* = 252.69	*D*_x_ = 1.387 Mg m^−^^3^
Monoclinic, *C*2/*c*	Mo *K*α radiation, λ = 0.71073 Å
Hall symbol: -C 2yc	Cell parameters from 3025 reflections
*a* = 16.7656 (4) Å	θ = 2.5–31.7°
*b* = 8.8171 (2) Å	µ = 0.31 mm^−^^1^
*c* = 17.0125 (4) Å	*T* = 293 K
β = 105.803 (1)°	Block, colourless
*V* = 2419.80 (10) Å^3^	0.30 × 0.25 × 0.20 mm
*Z* = 8	

### Data collection

**Table:**

Bruker Kappa APEXII area-detector diffractometer	4087 independent reflections
Radiation source: fine-focus sealed tube	2835 reflections with *I* > 2σ(*I*)
graphite	*R*_int_ = 0.026
ω and φ scans	θ_max_ = 31.7°, θ_min_ = 2.5°
Absorption correction: multi-scan (*SADABS*; Sheldrick, 2001)	*h* = −24→24
*T*_min_ = 0.912, *T*_max_ = 0.940	*k* = −13→12
17051 measured reflections	*l* = −25→23

### Refinement

**Table:**

Refinement on *F*^2^	Primary atom site location: structure-invariant direct methods
Least-squares matrix: full	Secondary atom site location: difference Fourier map
*R*[*F*^2^ > 2σ(*F*^2^)] = 0.043	Hydrogen site location: inferred from neighbouring sites
*wR*(*F*^2^) = 0.128	H atoms treated by a mixture of independent and constrained refinement
*S* = 1.02	*w* = 1/[σ^2^(*F*_o_^2^) + (0.0588*P*)^2^ + 0.9172*P*] where *P* = (*F*_o_^2^ + 2*F*_c_^2^)/3
4087 reflections	(Δ/σ)_max_ = 0.001
159 parameters	Δρ_max_ = 0.30 e Å^−^^3^
0 restraints	Δρ_min_ = −0.33 e Å^−^^3^

### Special details

**Table:**

Geometry. All e.s.d.'s (except the e.s.d. in the dihedral angle between two l.s. planes) are estimated using the full covariance matrix. The cell e.s.d.'s are taken into account individually in the estimation of e.s.d.'s in distances, angles and torsion angles; correlations between e.s.d.'s in cell parameters are only used when they are defined by crystal symmetry. An approximate (isotropic) treatment of cell e.s.d.'s is used for estimating e.s.d.'s involving l.s. planes.
Refinement. Refinement of *F*^2^ against ALL reflections. The weighted *R*-factor *wR* and goodness of fit *S* are based on *F*^2^, conventional *R*-factors *R* are based on *F*, with *F* set to zero for negative *F*^2^. The threshold expression of *F*^2^ > σ(*F*^2^) is used only for calculating *R*-factors(gt) *etc*. and is not relevant to the choice of reflections for refinement. *R*-factors based on *F*^2^ are statistically about twice as large as those based on *F*, and *R*- factors based on ALL data will be even larger.

### Fractional atomic coordinates and isotropic or equivalent isotropic displacement parameters (Å^2^)

**Table:**

	*x*	*y*	*z*	*U*_iso_*/*U*_eq_	
Cl1	0.44292 (4)	−0.22883 (6)	0.84052 (3)	0.07928 (19)	
O1	0.40338 (7)	−0.08925 (12)	0.45164 (6)	0.0519 (3)	
O2	0.31233 (7)	−0.25824 (11)	0.68948 (6)	0.0512 (3)	
N1	0.42899 (7)	0.08091 (13)	0.55371 (7)	0.0417 (2)	
H1	0.4810 (11)	0.0878 (19)	0.5523 (10)	0.052 (4)*	
C2	0.37704 (8)	−0.00571 (15)	0.49681 (7)	0.0406 (3)	
C3	0.28614 (8)	0.00659 (16)	0.48988 (8)	0.0422 (3)	
H3A	0.2551	−0.0382	0.4386	0.051*	
H3B	0.2711	0.1129	0.4890	0.051*	
C4	0.26188 (8)	−0.07178 (15)	0.55987 (8)	0.0413 (3)	
H4	0.2691	−0.1812	0.5543	0.050*	
N5	0.31771 (7)	−0.02397 (11)	0.63873 (6)	0.0373 (2)	
C6	0.34684 (8)	0.12893 (13)	0.64881 (7)	0.0361 (2)	
C7	0.31913 (9)	0.22890 (15)	0.69873 (8)	0.0439 (3)	
H7	0.2790	0.1982	0.7238	0.053*	
C8	0.35122 (10)	0.37389 (16)	0.71114 (9)	0.0497 (3)	
H8	0.3328	0.4406	0.7447	0.060*	
C9	0.41025 (10)	0.41982 (16)	0.67405 (9)	0.0503 (3)	
H9	0.4327	0.5167	0.6837	0.060*	
C10	0.43651 (9)	0.32312 (16)	0.62248 (9)	0.0452 (3)	
H10	0.4763	0.3552	0.5973	0.054*	
C11	0.40373 (7)	0.17797 (14)	0.60811 (7)	0.0364 (2)	
C12	0.17216 (10)	−0.0438 (2)	0.55670 (11)	0.0639 (4)	
H12A	0.1588	−0.0971	0.6007	0.096*	
H12B	0.1373	−0.0794	0.5055	0.096*	
H12C	0.1634	0.0629	0.5619	0.096*	
C13	0.33861 (8)	−0.12933 (14)	0.69903 (7)	0.0377 (3)	
C14	0.39552 (10)	−0.07538 (17)	0.77936 (8)	0.0502 (3)	
H14A	0.3639	−0.0165	0.8085	0.060*	
H14B	0.4379	−0.0100	0.7687	0.060*	

### Atomic displacement parameters (Å^2^)

**Table:**

	*U*^11^	*U*^22^	*U*^33^	*U*^12^	*U*^13^	*U*^23^
Cl1	0.1088 (4)	0.0608 (3)	0.0534 (3)	0.0146 (2)	−0.0032 (2)	0.00910 (19)
O1	0.0638 (6)	0.0553 (6)	0.0420 (5)	−0.0034 (5)	0.0236 (5)	−0.0139 (4)
O2	0.0711 (7)	0.0363 (5)	0.0469 (6)	−0.0094 (4)	0.0173 (5)	0.0016 (4)
N1	0.0455 (6)	0.0460 (6)	0.0362 (5)	−0.0005 (5)	0.0157 (4)	−0.0071 (4)
C2	0.0535 (7)	0.0411 (6)	0.0296 (6)	0.0012 (5)	0.0157 (5)	0.0000 (5)
C3	0.0510 (7)	0.0443 (7)	0.0297 (6)	0.0010 (5)	0.0084 (5)	−0.0011 (5)
C4	0.0514 (7)	0.0388 (6)	0.0329 (6)	−0.0055 (5)	0.0101 (5)	−0.0041 (5)
N5	0.0521 (6)	0.0314 (5)	0.0300 (5)	−0.0039 (4)	0.0139 (4)	−0.0040 (4)
C6	0.0473 (6)	0.0300 (5)	0.0317 (5)	0.0003 (4)	0.0121 (5)	−0.0033 (4)
C7	0.0547 (7)	0.0401 (7)	0.0403 (7)	0.0036 (5)	0.0189 (6)	−0.0069 (5)
C8	0.0658 (9)	0.0368 (7)	0.0450 (7)	0.0071 (6)	0.0123 (6)	−0.0112 (5)
C9	0.0611 (8)	0.0322 (6)	0.0517 (8)	−0.0038 (6)	0.0050 (6)	−0.0069 (6)
C10	0.0495 (7)	0.0398 (7)	0.0459 (7)	−0.0057 (5)	0.0122 (6)	−0.0007 (5)
C11	0.0430 (6)	0.0339 (6)	0.0315 (5)	0.0020 (5)	0.0090 (5)	−0.0024 (4)
C12	0.0541 (9)	0.0832 (12)	0.0549 (9)	−0.0114 (8)	0.0160 (7)	0.0011 (8)
C13	0.0491 (6)	0.0354 (6)	0.0335 (6)	−0.0010 (5)	0.0195 (5)	−0.0014 (4)
C14	0.0695 (9)	0.0445 (7)	0.0346 (6)	−0.0015 (6)	0.0108 (6)	0.0016 (5)

### Geometric parameters (Å, °)

**Table:**

Cl1—C14	1.7591 (15)	C6—C11	1.3913 (17)
O1—C2	1.2299 (15)	C7—C8	1.3808 (19)
O2—C13	1.2138 (15)	C7—H7	0.9300
N1—C2	1.3490 (17)	C8—C9	1.372 (2)
N1—C11	1.4077 (15)	C8—H8	0.9300
N1—H1	0.881 (18)	C9—C10	1.379 (2)
C2—C3	1.5002 (19)	C9—H9	0.9300
C3—C4	1.5247 (18)	C10—C11	1.3880 (18)
C3—H3A	0.9700	C10—H10	0.9300
C3—H3B	0.9700	C12—H12A	0.9600
C4—N5	1.4730 (16)	C12—H12B	0.9600
C4—C12	1.511 (2)	C12—H12C	0.9600
C4—H4	0.9800	C13—C14	1.5147 (19)
N5—C13	1.3573 (16)	C14—H14A	0.9700
N5—C6	1.4283 (15)	C14—H14B	0.9700
C6—C7	1.3888 (16)		
			
C2—N1—C11	124.40 (11)	C9—C8—C7	120.19 (13)
C2—N1—H1	118.0 (11)	C9—C8—H8	119.9
C11—N1—H1	116.9 (11)	C7—C8—H8	119.9
O1—C2—N1	121.06 (12)	C8—C9—C10	120.36 (13)
O1—C2—C3	121.61 (12)	C8—C9—H9	119.8
N1—C2—C3	117.32 (11)	C10—C9—H9	119.8
C2—C3—C4	112.78 (11)	C9—C10—C11	120.17 (13)
C2—C3—H3A	109.0	C9—C10—H10	119.9
C4—C3—H3A	109.0	C11—C10—H10	119.9
C2—C3—H3B	109.0	C10—C11—C6	119.41 (11)
C4—C3—H3B	109.0	C10—C11—N1	120.05 (12)
H3A—C3—H3B	107.8	C6—C11—N1	120.53 (11)
N5—C4—C12	111.43 (11)	C4—C12—H12A	109.5
N5—C4—C3	110.07 (10)	C4—C12—H12B	109.5
C12—C4—C3	111.88 (12)	H12A—C12—H12B	109.5
N5—C4—H4	107.8	C4—C12—H12C	109.5
C12—C4—H4	107.8	H12A—C12—H12C	109.5
C3—C4—H4	107.8	H12B—C12—H12C	109.5
C13—N5—C6	123.09 (10)	O2—C13—N5	122.02 (12)
C13—N5—C4	117.50 (10)	O2—C13—C14	122.09 (12)
C6—N5—C4	119.42 (10)	N5—C13—C14	115.88 (11)
C7—C6—C11	119.74 (11)	C13—C14—Cl1	111.36 (10)
C7—C6—N5	120.80 (11)	C13—C14—H14A	109.4
C11—C6—N5	119.46 (10)	Cl1—C14—H14A	109.4
C8—C7—C6	119.98 (13)	C13—C14—H14B	109.4
C8—C7—H7	120.0	Cl1—C14—H14B	109.4
C6—C7—H7	120.0	H14A—C14—H14B	108.0
			
C11—N1—C2—O1	−179.31 (12)	C7—C8—C9—C10	1.7 (2)
C11—N1—C2—C3	1.92 (19)	C8—C9—C10—C11	−0.4 (2)
O1—C2—C3—C4	107.11 (14)	C9—C10—C11—C6	−2.7 (2)
N1—C2—C3—C4	−74.12 (15)	C9—C10—C11—N1	178.28 (13)
C2—C3—C4—N5	49.39 (14)	C7—C6—C11—C10	4.55 (19)
C2—C3—C4—C12	173.86 (12)	N5—C6—C11—C10	−175.39 (12)
C12—C4—N5—C13	91.37 (15)	C7—C6—C11—N1	−176.42 (12)
C3—C4—N5—C13	−143.90 (11)	N5—C6—C11—N1	3.64 (18)
C12—C4—N5—C6	−88.25 (14)	C2—N1—C11—C10	−135.90 (14)
C3—C4—N5—C6	36.49 (15)	C2—N1—C11—C6	45.08 (18)
C13—N5—C6—C7	−69.95 (17)	C6—N5—C13—O2	179.08 (12)
C4—N5—C6—C7	109.65 (14)	C4—N5—C13—O2	−0.52 (18)
C13—N5—C6—C11	110.00 (14)	C6—N5—C13—C14	0.10 (18)
C4—N5—C6—C11	−70.41 (16)	C4—N5—C13—C14	−179.51 (11)
C11—C6—C7—C8	−3.4 (2)	O2—C13—C14—Cl1	19.10 (18)
N5—C6—C7—C8	176.59 (13)	N5—C13—C14—Cl1	−161.92 (10)
C6—C7—C8—C9	0.2 (2)		

### Hydrogen-bond geometry (Å, °)

**Table:**

*D*—H···*A*	*D*—H	H···*A*	*D*···*A*	*D*—H···*A*
C4—H4···O2	0.98	2.32	2.6952 (17)	102
N1—H1···O1^i^	0.881 (18)	1.958 (18)	2.8375 (16)	176.4 (16)
C7—H7···O2^ii^	0.93	2.43	3.2818 (17)	153
C14—H14A···O1^iii^	0.97	2.52	3.2411 (18)	131

Symmetry codes:  (i) −*x*+1, −*y*, −*z*+1; (ii) −*x*+1/2, *y*+1/2, −*z*+3/2; (iii) *x*, −*y*, *z*+1/2.
